# Accuracy of consumer-based activity trackers to measure and coach patients with lower limb lymphoedema

**DOI:** 10.1371/journal.pone.0305768

**Published:** 2024-07-18

**Authors:** Astrid Blondeel, Nele Devoogdt, Anne Asnong, Inge Geraerts, An De Groef, An-Kathleen Heroes, Charlotte Van Calster, Thierry Troosters, Heleen Demeyer, Pieter Ginis, Tessa De Vrieze

**Affiliations:** 1 Department of Rehabilitation Sciences, KU Leuven, Leuven, Belgium; 2 Department of Rehabilitation Sciences and Physiotherapy, MOVANT, University of Antwerp, Antwerp, Belgium; 3 Department of Rehabilitation Sciences, Ghent University, Ghent, Belgium; Helwan University Faculty of Engineering, EGYPT

## Abstract

**Purpose:**

This study investigated the accuracy of activity trackers in chronic lower limb lymphoedema (LLL) patients and in comparison to matched controls.

**Materials and methods:**

Seventeen LLL patients and 35 healthy subjects wore an activity tracker at the hip (Fitbit Zip/Inspire; hip-AT) and one at the wrist (Fitbit Alta/Inspire; wrist-AT) combined with a reference activity monitor (Dynaport Movemonitor; DAM), for 14 consecutive days. To analyze accuracy and agreement, mean daily step count from both AT’s were compared to DAM. To evaluate the accuracy as coaching tool, day-by-day differences were calculated. The Kendall correlation coefficient was used to test consistency of ranking daily steps between the AT’s and the DAM.

**Results:**

The wrist-AT significantly overestimated daily step count compared to DAM in the LLL group (+1221 ± 1754 steps per day, p = 0.011) while the hip-AT underestimated the step count, although not significantly. Similar results were found in the healthy control group. As a coaching tool, both wrist-AT and hip-AT showed a moderate correlation with the DAM (r = 0.507 and 0.622, respectively) in the LLL group regarding consistency of ranking from most to least active days.

**Conclusion:**

Wrist-AT’s significantly overestimate daily step count in a LLL population. As a coaching tool, both trackers show moderate validity, indicating applicability to improve physical activity.

## Introduction

Lymphoedema is a frequently occurring, chronic, debilitating disorder characterized by abnormal tissue swelling, adipose deposition and tissue fibrosis. It results from disruption, blockage, or genetic abnormalities of the lymphatic system whether or not in combination with increased filtration rates out of the blood system.

Patients with lower limb lymphedema (LLL) experience symptoms such as swelling and fibrosis that have a negative impact on a patient’s quality of life and daily functioning [1]. These problems in functioning also negatively influence a patient’s daily level of physical activity (PA) [2]. The odds of reporting poor physical functioning have been reported to be 5 times higher when diagnosed with LLL. However, these odds tend to decrease with higher levels of PA or more walking in daily life [2]. Also, higher levels of PA in these patients are associated with lower odds of reporting LLL symptoms, such as heaviness, general swelling, limb-related swelling, infection, aching, numbness and decreased function [3]. These findings highlight the importance of stimulating PA to both diminish LLL symptoms and to improve physical functioning in daily life. Additionally, the monitoring of PA in patients with LLL can be a valuable coaching tool to make patients aware of their decreased PA-level, to motivate them and to show them progress over time [4]. Subjective self-reported methods for measuring PA-levels (e.g. questionnaires, detailed diaries) might be subject to recall and/or response bias [5]. PA-levels can also be objectively measured using motion sensors such as pedometers, accelerometers and multiple-sensor devices [6].

Last years, usage of consumer-based hip-worn (*hip-AT*) or wrist-worn (*wrist-AT*) activity trackers has gained in popularity. Advantages of these devices are their ease of use and the lower cost when compared to medical device classified accelerometers. What might be helpful in clinical practice to promote the increase of daily steps, is the fact that they directly provide feedback regarding PA levels [7]. Depending on the type of device, activity-related parameters such as step count and energy expenditure are presented. Research has shown sufficient validity of previous devices of hip-worn activity trackers in healthy volunteers for step counts. However, an underestimation of the step count during slower walking has been demonstrated as well [8–10]. Newer generations of consumer-based activity trackers are available now but it remains unclear whether these activity trackers can be used as a valid way to measure PA both in research and in clinical practice, in patients with LLL. In addition, research showed that the wearing site of the tracker has an important impact on the measurement accuracy [11], although it is not known whether the placement of the tracker hip- or wrist-worn has an impact on the measurement properties of these wearables in patients with LLL. When aiming to use activity trackers as a physical activity coaching tool in patients with LLL, also user preference might play an important role in adherence to these devices. Up until now, this has not been investigated in this patient population.

Therefore, the first aim of this study was to investigate the accuracy of consumer-based activity trackers worn at the hip or wrist in patients with LLL to measure PA (in terms of daily step count) as compared to a valid activity monitor. These results in the LLL group are compared to results in an age-, gender- and BMI-matched healthy control group [12]. Secondly, validity of these consumer-based activity trackers as coaching tool (i.e. the accuracy to distinguish more and less active days at individual level), was investigated. Lastly, we aimed to provide insights into the user-friendliness of the wrist- and hip- AT based on the patient’s preferences.

## Materials and methods

This study was approved by the Ethical Committee of the University Hospitals Leuven (s60227). All participants signed written informed consent prior to data collection. This study is part of a series of studies on validity of consumer-based activity trackers conducted at the Department of Rehabilitation Sciences of KU Leuven in patients with non-communicable diseases (Parkinson’s disease [13], Chronic Obstructive Pulmonary Disease [7] and patients with breast or colorectal cancer [14]). As part of this project, an age-, gender- and BMI-matched healthy control group was available of which the results are published elsewhere [14].

### Study participants and design

In the present trial, patients with LLL were included. Patients were recruited in the Center for Lymphedema of the University Hospitals Leuven (Pellenberg, Belgium) between 01/08/2019 and 30/09/2021. Patients with a diagnosis of primary or secondary LLL, with stable stage 2a, 2b or 3 LLL according to International Society of Lymphology [15], were eligible for this study. Exclusion criteria were patients younger than 40, with cognitive impairments or unable to apply the activity trackers correctly. Subjects with morbid obesity (BMI > 40 kg/m^2^) or using a walking aid were also excluded, as well as people with a primary diagnosis of other chronic conditions that preclude normal physical activity. Additionally, data from 35 age-, gender- and and BMI-matched (i.e. similar age and gender distribution as the LLL population) healthy controls who were in absence of any known LLL was available for comparison. The inclusion criteria for the healthy subjects were no or marginal smoking history (<5 pack years). Exclusion criteria were the same as for the lymphoedema group.

### Procedure and outcomes

This prospective observational study consisted of one clinical assessment in the Center for Lymphedema and an data collection phase of 14 days at home. During this phase, participants were instructed to wear a ‘gold standard’ activity monitor (i.e. Dynaport Movemonitor) and two or three consumer-based activity trackers simultaneously, during waking hours. At the end of the data collection phase, a second clinical visit occurred in the center where participants were asked to fill in a questionnaire regarding the patient’s experience with wearing the activity trackers (i.e. user-friendliness).

At the first clinical visit, a clinical assessment was performed: 1) assessment of body composition (BMI based on weight and height); 2) a functional exercise capacity test by the best out of two six-minute walking tests (6MWT) conducted in a 50 m corridor and 30 minutes of rest in between both 6MWT [16]; and 3) symptoms of fatigue and dyspnea (both by BORG scale) at the end of the test [17]. Participant characteristics including age, work status and comorbidities were collected by self-report. Lymphoedema volume and severity stage were collected from the medical file of the patient as during the same week a consultation at the Center for Lymphoedema was scheduled as well, whereby limb volume was being measured, and the Lymph-ICF-LL questionnaire [18] regarding problems in functioning was completed.

**Fig 1:**
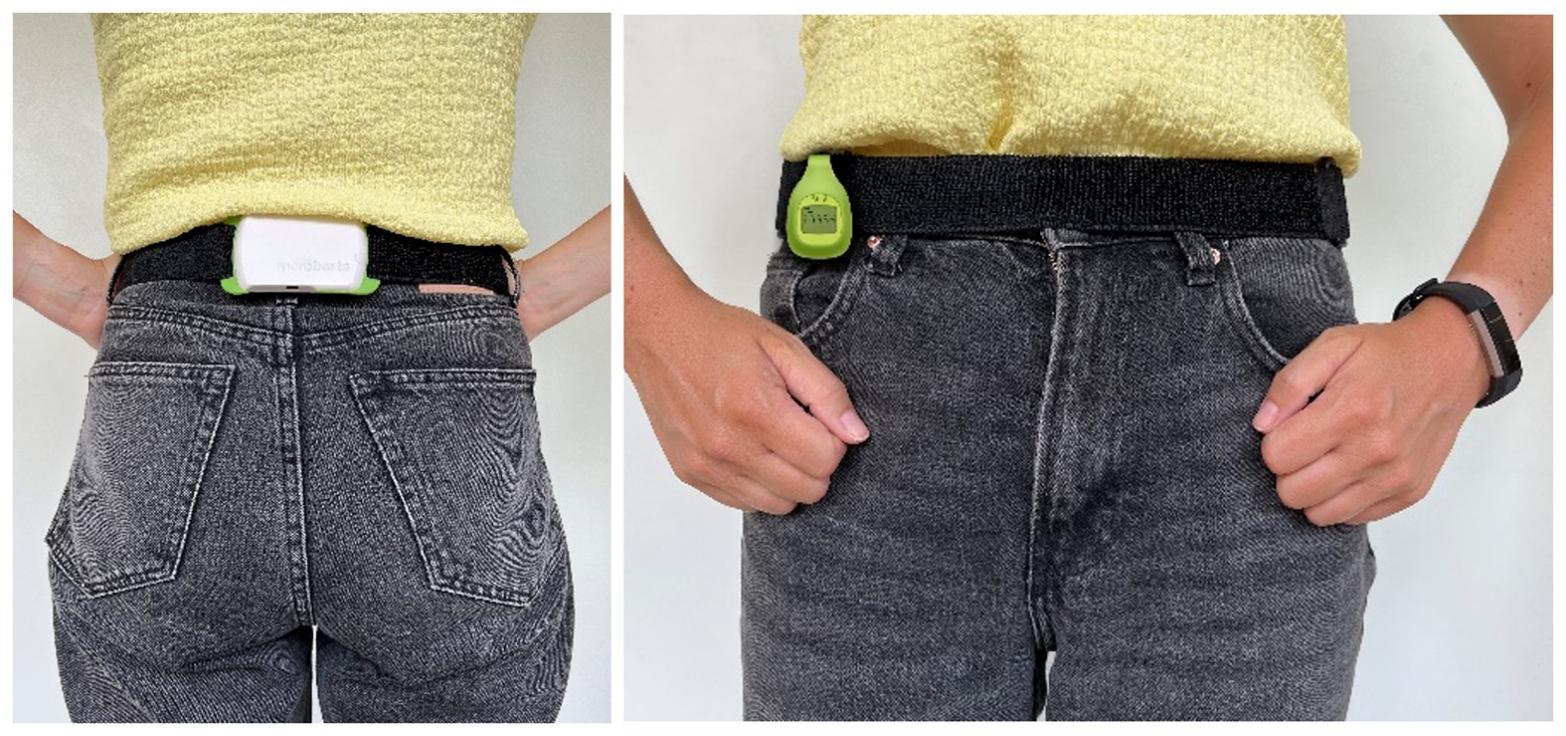
Example of the wearing procedure of the AT’s. Dynaport Movemonitor worn at the level of the hip (using a belt; device at the lower back). Hip-AT (Fitbit ZIP/Inspire) attached at the level of the hip using a clip. Wrist-AT (Fitbit Alta/Inspire) worn at the wrist.

Subjects received an activity monitor, i.e. **Dynaport Movemonitor** (DAM, McRoberts BV, The Hague, the Netherlands) and consumer-based activity trackers (Fitbit, Fitbit, Inc., San Francisco, USA) worn at the wrist (i.e. **Fitbit Alta** or **Fitbit Inspire**, generally classified as ‘wrist-AT’ throughout this paper) and worn at the hip (i.e. **Fitbit Zip** and/or **Fitbit Inspire**, generally classified as ‘hip-AT’), based on availability of the activity trackers (Fig 1). Subjects were instructed to simultaneously wear all devices for 14 consecutive days during waking hours, except for bathing, showering or swimming. The DAM contains a tri-axial accelerometer, which has already been validated to objectively measure PA in patients with non-communicable diseases (other than lymphoedema) such as COPD [19]. This device is worn at the lower back and does not provide direct feedback.

Similar to previous research [7], daily step count retrieved from the DAM, hip-AT and wrist-AT were used for the present analyses. Wearing time was registered by the DAM. Daily step counts of the activity trackers were extracted from the online Fitbit platform during the second visit at the hospital.

User-friendliness of the consumer-based wearables was evaluated through a self-developed questionnaire. Questions were 1) “How pleasant was it to wear this device?”; 2) “How often did you look at the steps displayed on the activity tracker?” and 3) “How long would you like to use this device in the future?”. Each question could be answered on a 5 point Likert scale. All data were entered in Redcap, a Research Electronic Data Capture System [20].

### Statistical analyses

Statistical analyses were performed using the IBM SPSS version 28.0.0.0 and SAS statistical package (V9.4, SAS Institute, Cary, North Carolina, USA). Data are presented as mean ± SD, unless stated otherwise. The Shapiro-Wilk tests revealed a normal distribution of data. Statistical significance was set at p<0.05 for all analyses. Subjects were included in the analyses if they had at least 3 valid days (i.e. *>* 8 hours of wearing time) with matching data with the other consumer-based devices. Statistical analyses were based on previous analyses performed by our research group, in a different population [7]. In case two hip-AT’s were worn (e.g. combinations 2 and 5, Fig 1), mean step count of the Fitbit Inspire (= newer device) was selected for analyses.

First, to investigate the accuracy of the consumer-based activity trackers to measure PA, we 1) compared mean daily step count per patient measured by hip-AT and wrist-AT with DAM (= reference, gold standard), by use of a paired t-test; 2) we compared mean daily step count measured by each type of device between the lymphoedema group and healthy control group using an unpaired t-test; and 3) we analyzed the agreement of the mean step count measured by hip-AT and wrist AT as compared to the DAM using Intraclass Correlation Coefficients (ICC’s) (two-way mixed with absolute agreement) and Bland-Altman Plots in the LLL group.

Second, to evaluate the accuracy of these activity trackers as coaching device for an individual, first, day-by-day differences to the individual mean PA (in terms of step counts) were calculated. This was done for each patient for each of the devices (DAM, hip-AT and wrist-AT). If more than 10 valid days were available, this analysis was based on 10 randomly selected days. Else, all available valid days were used. The day-by-day data were individually sorted based on the DAM measurement, from most active day to least active day and corresponding days with hip-AT and wrist-AT were added to the sorted database. Mean day-by-day differences for each day for each device were calculated and graphically presented. Additionally, the step count data retrieved from the three devices were used to evaluate consistency of ranking between DAM and hip-AT/wrist-AT. Daily step count of the selected 10 random days were ranked from most active day to least active day for each device separately. The ranking scores of each day for each device was compared. Consistency of these rankings was evaluated by a Kendall correlation. Correlation coefficient (for both ICC’s as well as Kendall correlations) was interpreted using the following cutoffs: weak correlation r = 0.30–0.50; moderate correlation r0.51–0.70; strong correlation r = 0.71–0.90; very strong correlation r*>*0.90 [21].

Finally, user friendliness and preference of the trackers was presented descriptively.

## Results

In total, 21 patients signed informed consent. However, data of 4 patients was not suitable for analysis as there were too much missing data for several (technical) reasons (malfunctioning or forgetting to charge the devices). Consequently, data from 17 patients (mean (SD) 11.6 (3.2) days of wearing, total of 185 patient-days) with LLL were included in the final analyses. In addition, data from 35 age-, gender- and BMI-matched healthy controls (mean (SD) 13.2 (2) days of wearing, total of 462 patient-days) was available for comparison [14]. Thirty-eight percent of patients had an average step count of less 5000 steps with DAM, 81% patients had less than 7500 steps per day. These proportions were significantly higher than in healthy subjects (14% and 43% respectively, p<0.05). Patients also had a lower functional exercise capacity compared to the control group. Subject characteristics are presented in Table 1.

**Table 1:** Characteristics of all subjects, expressed as mean ± standard deviation and lymphoedema-specific characteristics (LLL group, n = 17), expressed as mean ± standard deviation (except when indicated with ◼, median ± inter quartile range are reported).

**General participant characteristics**					
	n	Lymphoedema group	n	Healthy control group	p-value
Age (years)	17	58 ± 13	35	58 ± 6	0.859
BMI (kg/m^2^)	17	28.7 ± 5.4	35	26.2 ± 3.9	0.068
6MWD (m)	17	436 ± 112	29	625 ± 68	< .001
6MWD (% pred)	17	67 ± 15	29	91 ± 12	< .001
Gender					
% Female	9	53	19	54	
% Male	8	47	16	46	
**Lymphoedema-specific characteristics**			
		Lymphoedema group	
Duration lymphoedema (months)◼		44 ± 59	
Total score Lymph-ICF-LL*		33 ± 19	
Physical functioning		33 ± 18	
Mental functioning		32 ± 26	
Household activities		21 ± 21	
Mobility activities		38 ± 26	
Social life		41 ± 29	
Excessive lymphoedema volume (ml)		1160.7 ± 842.2	
Location lymphoedema			
% Unilateral		53	
% Bilateral		47	
Lymphoedema stage (%)**		
% Stage 2a		82
% Stage 2b		18
% Stage 3		0

* The Lymph-ICF-LL total score represents the amount of problems in daily functioning (physical functions, mental functions, mobility activities, household chores and participation in social life) due to the lower limb lymphoedema.

** According to the ISL severity scale [15]. P-value based on independent t-test or Chi-Square. BMI = Body Mass Index, 6MWD = 6-minute walking distance [22].

### Accuracy of the consumer-based activity trackers to measure PA

In the LLL group, the mean daily step count recorded with a wrist-AT was significantly higher compared to the DAM, with a mean (SD) difference of +1221 (1754) steps per day or +22%; p = 0.011 (Table 2). Mean daily step count recorded with a hip-AT was not significantly different from the DAM. In the healthy control group, reported elsewhere, similar findings were found [14]. The mean (SD) daily step count was significantly lower in the patients with LLL compared to the healthy controls, measured with the wrist-AT (-3660 (829) steps per day; p<0.001), the hip-AT (-2957 (783) steps per day; p<0.001) and the DAM (-3289 (993) steps per day; p = 0.002).

**Table 2:** Mean ± standard deviation (SD) daily step count in lymphoedema. P-value compares mean step count measured by Dynaport Movemonitor (DAM) to respectively wrist-AT and hip-AT.

	n	DAM	Wrist-AT	Hip-AT
Average daily step count lymphoedema group (mean ± SD)	17	5504 ± 2467	6728 ± 2223	5177 ± 2192
	Δ	-	1221 ± 1754	-331 ± 1117
			**p = 0.011**	**p = 0.239**

AT = activity tracker; DAM = Dynaport Movemonitor Δ mean bias (*± SD step difference)* when compared to DAM and p-value

As shown in Table 3, DAM showed a very strong agreement, expressed as the ICC, with the hip-AT and a strong agreement with wrist-AT.

**Table 3:** Agreement of the mean step count measured by hip-AT and wrist AT as compared to the DAM using Intraclass Correlation Coefficients (ICC’s) (two-way mixed with absolute agreement).

		Wrist-AT	Hip-AT
		ICC (95% CI)	ICC (95% CI)
Lymphoedema group	DAM	0.784 (0.306–0.926)	0.938 (0.831–0.977)
		(n = 17)	(n = 17)

AT = activity tracker; DAM = Dynaport Movemonitor.

**Fig 2:**
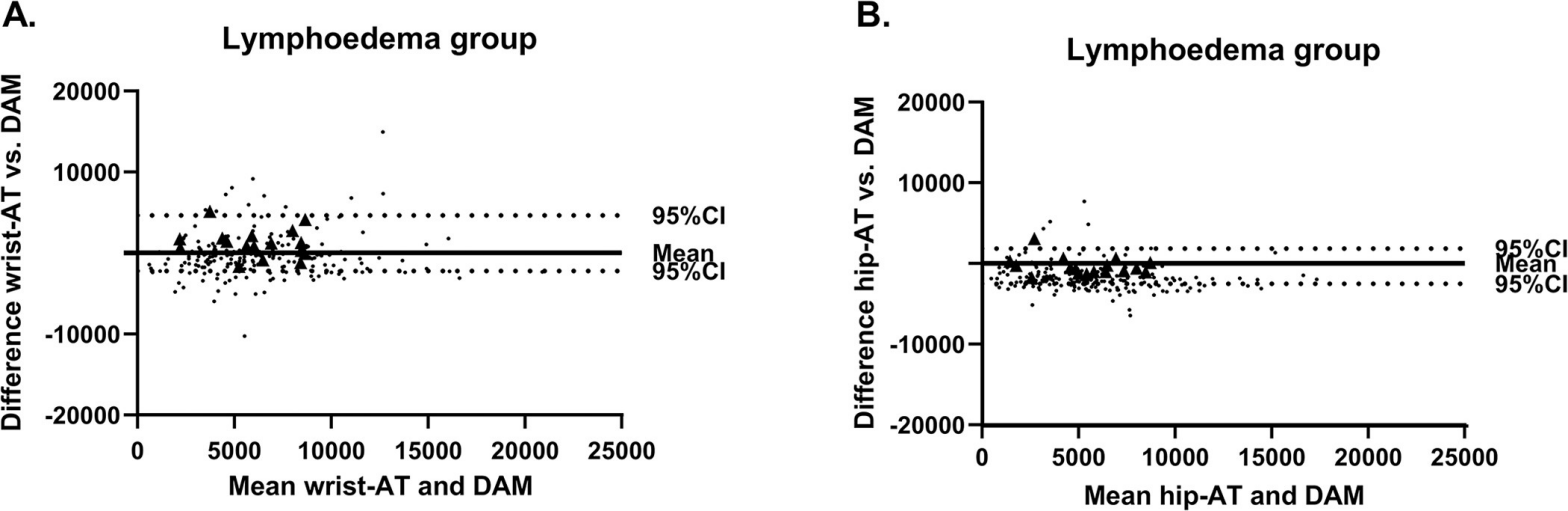
Bland-Altman plots with mean and 95%CI for the wrist-AT and hip-AT compared to DAM. Triangle symbols represent the mean individual step count per subject (n = 16); dots represent the daily step count. Means and 95%CI are calculated based on mean daily step count data. AT = activity tracker; DAM = Dynaport Movemonitor.

In Fig 2, the Bland Altman analysis is presented. Regarding the wrist-AT, the plot shows a mean bias (versus DAM) of 1221 (-2216;4657) steps. The mean bias for the hip-AT was -331 (-2522;1859) steps.

### Accuracy of these activity trackers as coaching device for an individual

**Fig 3:**
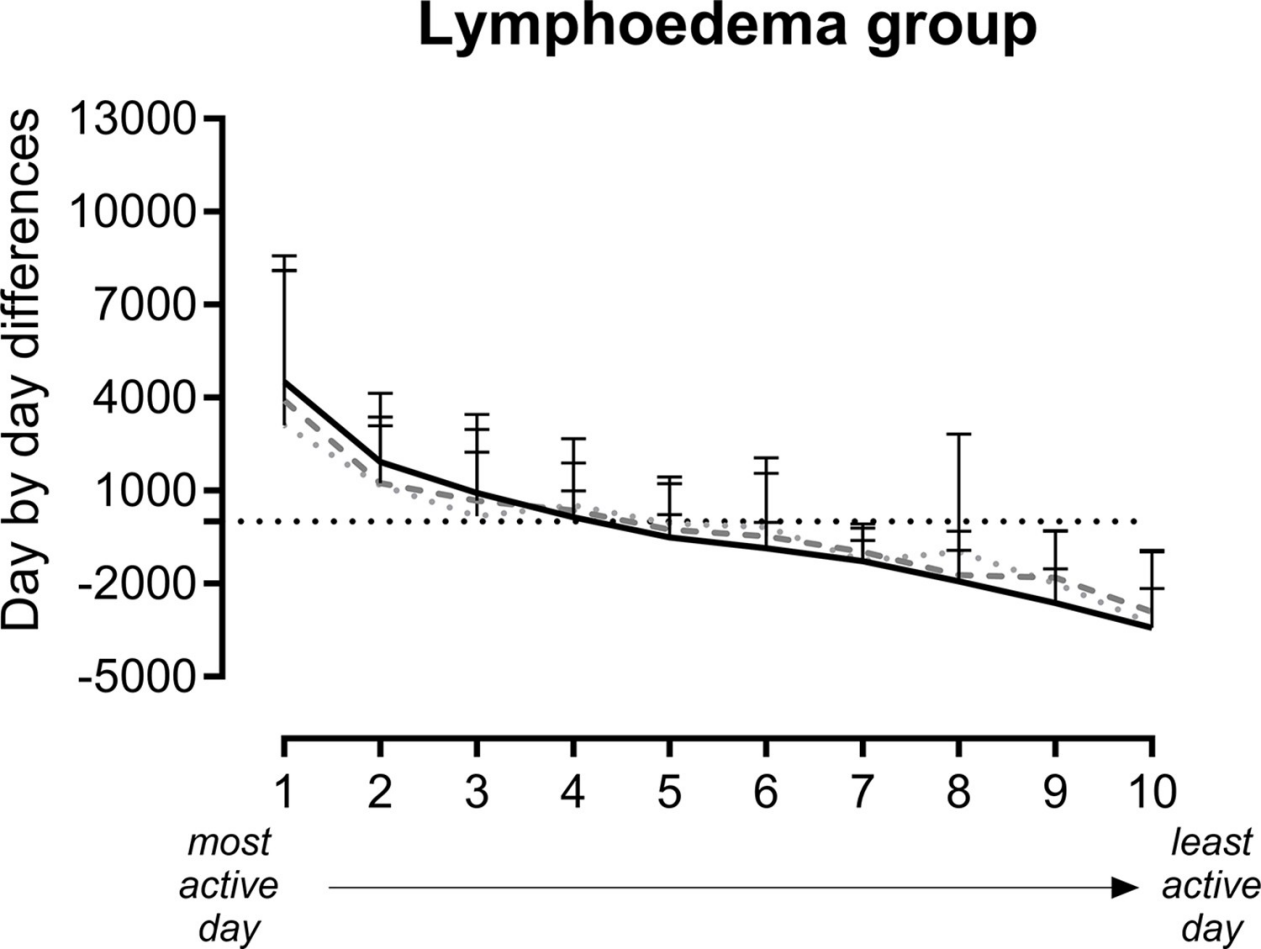
Graphical representation of day-by-day variability in steps per day around the individual mean step count. Mean (SD) day-by-day difference around the mean, measured with the hip-AT, wrist-AT and DAM. Horizontal line represents the mean step count, positive numbers represent more active days compared to the mean PA, negative numbers represent less active days. Days are ranked from most active to least active days according to DAM. AT = activity tracker; DAM = Dynaport Movemonitor.

**Fig 4:**
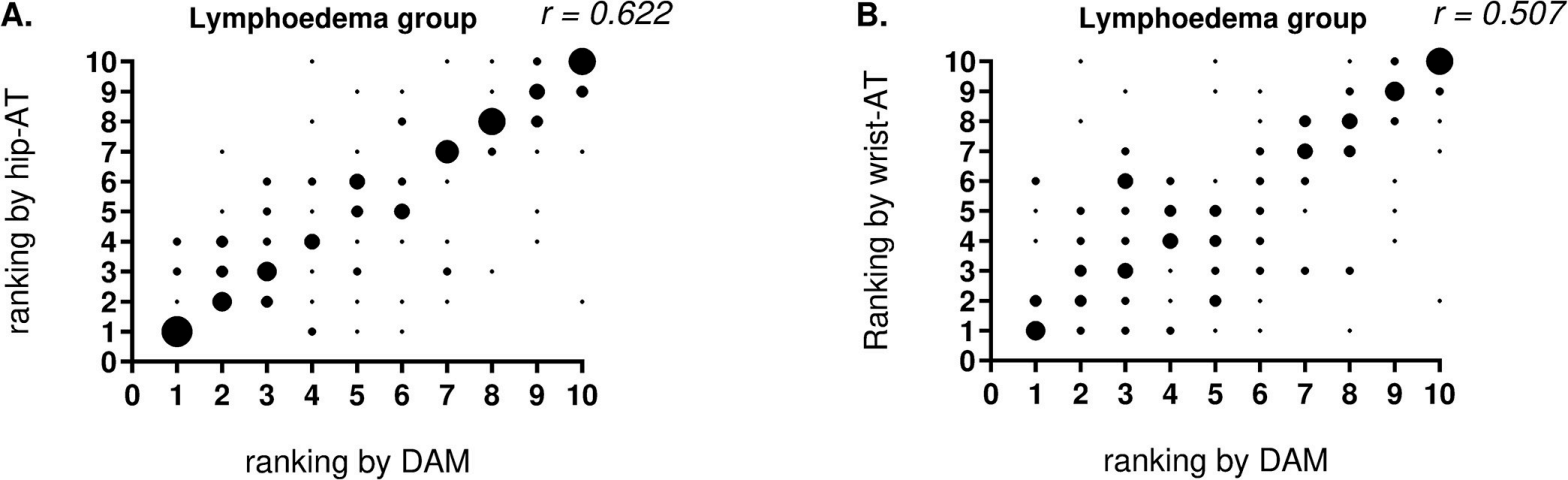
Ranking of daily step count by DAM compared to ranking by the AT’s. DAM compared to hip-AT (A) and compared to ranking by wrist-AT (B). The larger the dot, the larger the number of subjects for the given combination of ranks; r = Kendall correlation for consistency of ranking. AT = activity tracker; DAM = Dynaport Movemonitor.

Fig 3 displays the day-by-day variability expressed as mean day-by-day differences as recorded by the three devices. Visual evaluation of the plots shows that differences to the individual mean step count display the same pattern measured by the reference activity monitor (DAM), the hip-AT and the wrist-AT. This implicates that both consumer-based activity trackers were able to detect patterns of more and less active days, similarly as with the reference activity monitor (DAM). Similar results were found in the healthy control group [14]. In addition, a moderate Kendall correlation coefficient (as measure of consistency of ranking form most active to less active days) was found for the wrist-AT and hip-AT, in the lymphoedema group (Fig 4). In the healthy control group, a moderate correlation coefficient was found for both the wrist-AT and hip-AT [14].

### User friendliness of the trackers

Regarding user preferences (Table 4), data of only 41% (wrist-AT) and 53% (hip-AT) lymphoedema patients was available. No clear difference regarding preference to wear either a wrist-AT or hip-AT was present is this group. Around 43% found it pleasant or very pleasant to wear either a wrist-AT or a hip-AT, all other patients remained neutral. No one indicated wearing an AT to be unpleasant. Answers from the LLL group were comparable to these of the healthy controls [14].

**Table 4:** User preference in %.

		Lymphoedema group	
		Hip-AT (9 or 8° responders)	Wrist-AT (7 responders)
**How pleasant was it to wear the wearable?**	Very pleasant/ pleasant (%)	44.4	42.9
	Neutral (%)	44.4	57.1
	Not pleasant (%)	0.0	0.0
**How many times did you look at your step count?**	Multiple times a day (%)	66.7	71.4
	Once a day (%)	0.0	0.0
	Once or twice a week (%)	22.2	28.6
	Never (%)	11.1	0.0
**How long would you like to wear the wearable in the future?°**	A year (%)	11.1	14.3
	Months (%)	22.2	0.0
	Weeks / days (%)	44.4	71.4
	Never (%)	12.5	14.3

In the lymphoedema group, 14% would wear a wrist-AT for more than a year, versus 11% for the hip-AT. In the healthy control group, 47% and 17% would wear a wrist- or hip-AT, respectively, more than one year [14]. Detailed information about the user preferences are provided in Table 4.

## Discussion

As previous research on measurement properties of consumer-based activity trackers in patients with LLL was currently missing, we are the first to provide evidence on accuracy of consumer-based activity tracker in patients with LLL. Low physical activity levels in these patients, confirmed in the present study, and the possibility to use consumer-based trackers to coach patients warrant better knowledge on their usefulness. Therefore, the aim of this study was to investigate the accuracy of consumer-based activity trackers worn at the wrist and hip for measuring step count during daily life in patients with chronic LLL and healthy age-, BMI- and gender-matched controls. In addition, validity of these wearables as coaching tool, i.e. the accuracy to differentiate between more and less active days at an individual level, was investigated. Lastly, user friendliness of these devices was explored.

Although patients had a significant lower step count with all three types of wearables (wrist-AT, hip-AT and DAM), results showed that wrist-AT’s significantly overestimate step count both in patients with LLL as in healthy controls [14]. Hip-AT’s underestimated daily step count, albeit not statistically significant. Earlier research stated that the difference between the daily steps measured by the activity tracker and the gold standard (DAM) should not exceed 10% in a daily living situation [23]. In the present study, the wrist-AT’s reached this cut-off of 10% in both populations, questioning their **accuracy as measurement device**. In contrast, the hip-AT’s did not reach the 10% cut-off in both groups. These findings are confirmed by only a poor agreement between wrist-AT and DAM, which was more pronounced in healthy (i.e. more active) subjects [14]. Results of this paper are in line with other studies. Although a lot of research on clinimetrics exists in healthy subjects as well as in different specific populations such as patients with chronic pain [24], COPD [7, 25], prostate cancer [26, 27], multiple sclerosis [28] or cardiac diseases [29], findings based on patients with chronic LLL is missing. Alternatively, validity of consumer-based activity trackers in people living with a chronic disease in general [30], or in older, community-dwelling adults [31] has been investigated. An overestimation by wrist-AT’s is commonly reported and can be attributed to upper limb movements not directly related to walking that are oftentimes registered as steps [11, 32]. At the hip, a recent study showed that, despite an excellent agreement, there was a significant undercounting of steps by the Fitbit Zip (attached to the shoe) compared to manual count in a mixed subacute rehabilitation population [10]. Additionally, the accuracy of the Fitbit Zip was reduced in those who walked slower (<0.8m/s or 3km/h on a treadmill) [10, 33]. Also it was demonstrated that Fitbit wearables were most accurate when walking on a treadmill and least accurate during walking with a walking aid and at low walking speed. This may warrant cautious interpretation of step counts in LLL patients using assistive devices (if applicable) [11].

Although the LLL group was age-, BMI- and gender-matched with the healthy controls, showing a non-significant difference in BMI, there was a significant lower functional exercise capacity in patients with LLL. In line, mean step count was significantly lower in the LLL group compared to the healthy control group. Outcomes from the Lymph-ICF-LL questionnaire showed that patients experienced only minor problems regarding household chores (mean ± SD subscore 21±21) and moderate problems in mobility (mean ± SD subscore 38±26) in daily life. Nevertheless, literature shows that approximately 7000 to 8000 steps/day is a reasonable threshold of free-living PA that is also associated with current public health guidelines’ emphasis on minimal amounts of time spent in moderate-to-vigorous PA [34]. As the mean step count in the lymphoedema group was only 5504 steps/day, interventions aiming to increase PA in patients with LLL are needed. In this, the use of activity trackers as coaching tool providing direct feedback can be helpful [35].

In order to be used as input in physical activity coaching, both wrist-AT and hip-AT should be able to distinguish more and less active days on an individual day-by-day level. Kendall plots were somewhat less strong compared to similar plots obtained in patients with COPD, hence some caution is perhaps needed to use wrist worn AT’s in patient with LLL. Therefore, these consumer based AT’s seem nevertheless valid to be used as a coaching tool at an individual participant level. Studies using AT’s to improve adherence and motivation in patients with LLL are needed. In cancer survivors, promising results are shown in improving a wide range of health-related outcome, such as anxiety, fatigue and depression [36]. In those studies, a common approach is seen in the fact that the AT is used as a coaching tool rather than a measurement device (e.g. in combination with a smartphone application or with remote monitoring by a healthcare provider) [36]. In patients with LLL, findings of the present study support the use of these devices as a coaching device in improving daily PA, although a surprisingly low proportion of patients would agree to wear AT’s for a long period.

**User friendliness** of the devices was also investigated. Wrist-worn devices might be more convenient for direct feedback on PA, more comfortable and less embarrassing to wear with overall high acceptability [7, 37–39]. In the present study, wrist-AT’s were preferred by healthy controls as well [14], however, no clear preference between wrist-AT and hip-AT could be noticed in the lymphoedema group. When choosing an AT, also education level and aesthetics (including size) have been reported to be important factors [37, 40]. To stimulate PA and to achieve a long-term change in health behaviour, motivation, starting with wearing an AT, is one of the most important aspects [41, 42]. As was noticed that in the present study, only 1 out of 4 (or 25%) responders (both groups together) would be willing to wear an AT for longer than a year, it is of utmost importance to promote wearing an AT in order to make it more naturally and accessible in older lymphoedema patients as well. Importantly, as there was a lot of missing data regarding user friendliness of the devices in the lymphoedema group, one should be attentive to any possible bias when interpreting these outcomes.

The strength of this study is the application of specific statistical approaches to examine the accuracy of consumer-based AT’s both as a *measurement* device and as a *coaching* device. This is the first study examining these aspects in patients with LLL. Additionally, a data collection period of 14 days in the daily living context was incorporated. Some limitations should be mentioned as well. Firstly, our lymphoedema group consisted of only 17 subjects. Therefore, results should be interpreted with caution. Although recorded as an inclusion criterion, no patients with lymphoedema stage 3 were included, limiting our findings to be generalized to this patient group as well. Nevertheless, our sample size was based on comparable studies in the field in other patient-groups with non-communicable diseases [7] and should be large enough to cover the whole spectrum of lymphoedema stage II (IIa and IIb) which are most common. Additionally, by comparing the results with a healthy age-, BMI- and gender-matched control group and by testing in free-living conditions, the external validity of our results increases considerably. Secondly, the DAM served as the gold standard monitor, allowing testing in free-living conditions. However, video recording might be an even more valid reference criterion. Thirdly, several wearables from Fitbit were used. If possible, the most recently released device was selected for analyses over older ones. To demonstrate interchangeability of devices, a sensitivity analyses among older and newer devices worn at the same location was performed at our research group [14]. We found very small differences which might also be influenced by the wearing of the devices, which we cannot control as no information on wearing time is available for the trackers individually.

However, technology keeps improving and adapting fast with frequently new releases on the market. Therefore, caution is warranted when translating our results to other (newer) devices of Fitbit and other brands. However, as our results are in line with previous studies involving other brands and models of AT’s, they can be considered cautiously generalizable [7].

### Clinical implications and future research

This study shows that consumer-based activity trackers can be used in coaching interventions for patients with LLL to promote physical activity. Future trials can incorporate these activity trackers to provide real-time feedback on physical activity behavior, combined with well-designed behavior-change interventions, to stimulate participants towards a healthier lifestyle. Further investigation is needed aiming for effective strategies towards physical activity behavior change in patients with LLL, on the short as well as long-term.

Long-term compliance and adherence to activity trackers could be an important bottleneck when aiming to use these devices in long-term coaching interventions. To improve compliance, patient involvement is needed to choose the device that fits the needs of its user (location of wearing the device, look-and-feel, size, …) and therefore it can be recommended to include multiple device options in clinical practice and future trials.

Although generalizabilty of our results among different brands of consumer-based activity trackers is limited [43], the differences in steps measured by the wrist- or hip-worn device shown in our study are similar to previous research [7, 12, 13, 44, 45], confirming that the location of wearing the device plays a major role, independent of the brand. Yet, as technological advances in the field of activity trackers keeps growing, validation of newer devices in the specific target population (i.e. healthy or chronic diseased) remains warranted.

## Conclusion

In contrast to hip-worn devices, wrist-worn wearables significantly overestimate daily step count, both in patients with LLL and in healthy controls [14] and can therefore not be used as an accurate outcome measure for physical activity. However, our data suggest that these consumer based activity trackers can be used as a coaching tool to motivate PA. Both a wrist-AT and a hip-AT (based on consumer preference) seem feasible, consistent and thereby a valid tool on an individual level in patients with LLL.

### Implications for rehabilitation

- Consumer-based activity trackers (AT’s) are suggested to evaluate and stimulate physical activity. Nevertheless, the accuracy of these trackers is unknown in lower limb lymphoedema (LLL) patients.
- Wrist-AT’s significantly overestimate daily step count in a LLL population.
- Both a wrist-AT and a hip-AT (based on consumer preference) seem feasible, consistent and thereby a valid tool on an individual level in patients with LLL to motivate or improve physical activity.
